# Tuberculous Pyomyositis: A Rare but Serious Diagnosis

**DOI:** 10.1155/2013/126952

**Published:** 2013-03-24

**Authors:** Vikram Krishnasamy, Matthew Joseph

**Affiliations:** UPMC Montefiore Hospital, N-715, 200 Lothrop Street, Pittsburgh, PA 15213, USA

## Abstract

Tuberculous pyomyositis is a rare clinical entity with serious consequences if a diagnosis is not established early. A 53-year-old female with a past medical history of sarcoidosis and pulmonary fibrosis presented from an outside hospital with persistent fevers and a rash. She had been hospitalized multiple times at an outside hospital without any improvement in her symptoms. On examination, she was noted to have a large area of left upper lower extremity (LUE) tenderness with superimposed erythema. Laboratory data revealed a white blood cell count of 22,300. Computed tomography (CT) scans of the LUE, chest, and left lower extremity (LLE) showed multiple intramuscular abscesses in those regions without evidence of osteomyelitis. Subsequent drainage of the abscesses and resulting cultures revealed *Mycobacterium tuberculosis*. The patient was started on therapy with rifampin, isoniazid, pyrazinamide, and ethambutol. However, the patient developed hepatitis on these agents and subsequently went into septic shock with multiorgan failure. Care was eventually withdrawn as a result of a poor prognosis. This case illustrates the severe consequences of TB pyomyositis if not diagnosed promptly. While tuberculosis is uncommon in the United States, it should be an important consideration in the differential diagnosis of immunocompromised patients.

## 1. Case Presentation

The patient is a 53-year-old Caucasian female with a history of sarcoidosis and pulmonary fibrosis on chronic glucocorticoids who presented to the hospital with a 6–8 month history of progressive pain, erythema, and edema on the left scapula, biceps, and groin. The symptoms started with a burning sensation over her left scapula. Her husband noted at the time that she had a furuncular lesion in the area. Of note, there was no prior trauma to the area. The lesion subsequently progressed in size and pain, and she then developed fevers, chills, and a home recorded temperature of 102°F. She also progressed to left medial arm pain, swelling, and redness as well as left leg erythema and pain. On review of systems, she endorsed subjective fevers, chills, and night sweats; a two-month history of blurry vision; baseline dyspnea on supplemental oxygen use of 4 liters by nasal cannula; and a recent sinus infection. She denied any cough, wheezes, chest pain, palpitations, nausea, vomiting, abdominal pain, or dysuria. She was hospitalized multiple times at an outside hospital (OSH) where she received vancomycin, meropenem, and doxycycline. Due to progression of her illness despite treatment, she was subsequently transferred to our hospital.

Her past medical history included sarcoidosis and pulmonary fibrosis for which she was on 10 mg bid of prednisone for about one year. Her only surgical history was a cholecystectomy. Her medications prior to admission included fentanyl, prednisone, furosemide, fluoxetine, metoprolol, and ferrous sulfate. Significant allergies included sulfa and penicillins both of which caused hives. She had a 10-pack-year history of tobacco use, but her last cigarette was 25 years ago. Her past occupation was a secretary, but she was retired at the time of presentation. She currently resides with her husband in a house located in a wooded area of western Pennsylvania. Her travel history was as far north as New York and as far south as South Carolina. There was no recent travel history. She has 3 pets: 2 dogs and 1 cat. There was no significant family history of tuberculosis. It is unknown if she ever had a tuberculin skin test. 

**Figure 1:**
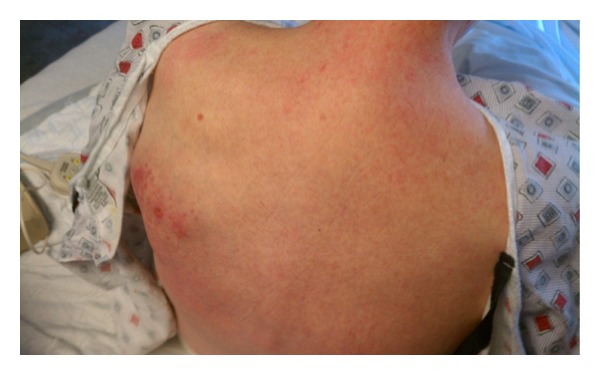
Focal area of involvement on posterior chest wall.

On initial physical exam, the patient had a temperature of 36.2°C, blood pressure of 110/62 mmHg, pulse of 78 beats/min, respiratory rate of 18 breaths/min, and an oxygen saturation of 98% on 4 liters of supplemental nasal oxygen. She was toxic appearing, and in pain. Her pupils were equal, round, and reactive to light. Extraocular movements were also intact. The neck was supple, without thyromegaly or jugular venous distension. Her lungs were clear to auscultation bilaterally, and on cardiac exam, she had regular heart sounds. The abdomen was soft, nontender, nondistended, and without hepatosplenomegaly. On her left upper back, she had a well-defined boggy area of tissue which was mildly erythematous and tender to palpation. Within the boggy area was a 5 mm hollow, circular ulceration without fluctuance or purulence (Figure 1). On her left arm, she had erythema, purpura, and induration which were painful to palpation. On her LLE, she had 2+ pitting edema and erythema extending from the groin to the foot. There was no cervical, axillary, epitrochlear, or inguinal lymphadenopathy. 

Initial laboratory values are summarized in Table 1.

## 2. Hospital Course 

**Figure 2:**
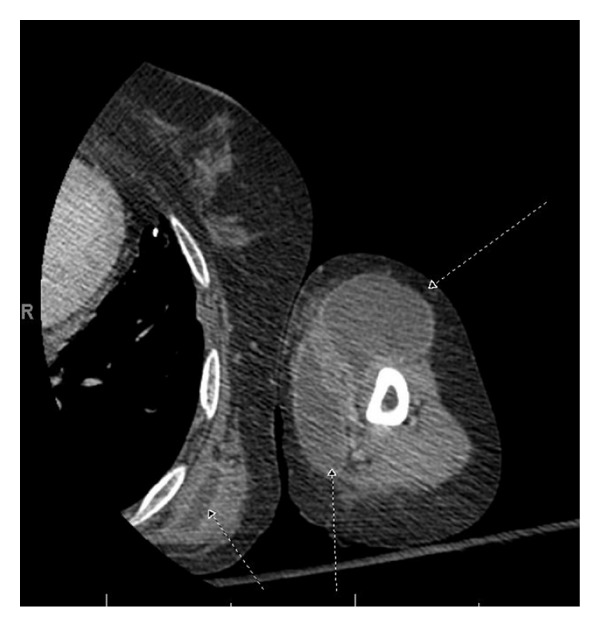
Multiple abscesses in the left upper extremity and chest wall.

The patient was admitted to the general medicine service and started on empiric broad spectrum antibiotics. CT scans of the left arm and left leg revealed multiple subcutaneous and intramuscular abscesses in the region of the left shoulder, left arm, and left chest wall without evidence of osteomyelitis (Figure 2). A large intramuscular abscess in the anterior compartment of the left thigh approximately 12 cm in diameter was also found. A subsequent CT of the chest, abdomen, and pelvis demonstrated ground glass opacities in the right upper lung and bilateral lower lungs, centrilobular nodules, a right upper lobe calcified granuloma, prominent paratracheal and anteroposterior window lymph nodes, steatosis of the liver, and parts of the fluid collections in the thigh as noted above. She was started on empiric antibiotic therapy with vancomycin, ceftriaxone, and metronidazole pending drainage of the sample with cultures and sensitivities. She then developed a rash on vancomycin and was switched to linezolid. 

 Interventional radiology was consulted to biopsy the affected areas. Multiple attempts were made in the back, left arm, and left inguinal region with the only successful aspiration from the left inguinal region. The other sites failed due to the thickness of the aspirate. The aspirate showed acid-fast bacilli on smear and subsequently *Mycobacterium tuberculosis* (MTB) on hospital day 14 via DNA probe. At that time, empiric antibiotics were stopped, and quadruple antituberculin therapy was initiated with isoniazid/pyridoxine, rifampin, pyrazinamide, and ethambutol. 

Concurrently, given the ground glass opacities and lack of cough or sputum production by the patient, pulmonology was consulted for bronchoscopy and bronchoalveolar lavage (BAL) to rule out pulmonary tuberculosis. One month after bronchoscopy, the culture from the BAL turned positive for MTB. 

There were concerns about the patient's potential wound healing given the degree of tissue involved as well as chronic steroid use. As a result, surgical debridement was deferred. Unfortunately, on hospital day 28, the patient developed elevated liver function tests (LFTs) necessitating cessation of rifampin, isoniazid, and pyrazinamide. Moxifloxacin was added as a second-line agent. Once her LFTs normalized, rifampin was added. However, several days later, the patient developed fevers and diarrhea. She was found to be hypotensive with a systolic blood pressure in the 70s and was transferred to the intensive care unit. She was started on vasopressors for septic shock, intubated for respiratory failure, and started on continuous renal replacement therapy for acute kidney injury. Over the ensuing days, given her overall poor prognosis, care was deescalated and the patient died. 

## 3. Discussion

Pyomyositis is a purulent infection of muscle that is generally the result of hematogenous spread [1]. Typically, the infection is known to occur in the tropics in otherwise healthy individuals with no comorbidities. Those afflicted in more temperate regions tend to have severe underlying comorbidities or are immunocompromised in some way [2]. 

The most common organisms implicated in pyomyositis include *Staphylococcus aureus* as well as, increasingly, MRSA. Group A streptococci are also common with gram-negative bacilli and pneumococci, with non-group A streptococci occurring less often [2]. There have also been instances of mycobacterial-induced pyomyositis [3]. 

Typical presentations of pyomyositis involve fever and pain localized to a muscle group, generally the lower extremities [2]. The infection usually progresses in three stages. Stage one tends to be characterized by fever, muscle pain, and swelling. Since this is an early stage, an abscess may not yet be apparent, and very few patients present at this stage. Stage two is where most patients present and occurs 10–21 days after initial symptoms begin. It is characterized by fever, muscle tenderness, and leukocytosis. Aspiration of the area will yield purulent material. Finally, stage three is the most severe and is accompanied by systemic toxicity. As a result, patients can develop complications of bacteremia [4]. One study showed a mortality rate that reached ten percent [5]. 

Diagnosis is typically made by radiography, predominantly by CT, and culture data [6]. MRI may also be helpful, especially in tuberculous pyomyositis [7, 8]. Because pyomyositis arises from hematologic spread, cultures, both from blood and drainage specimens, are extremely useful for determining appropriate antibiotic use [1, 2]. 

Treatment is dependent on the stage of the disease with stage 1 able to be treated by antibiotics. Stages 2-3 require both drainage as well as antibiotic treatment. Drainage is typically CT guided, but in the face of extensive disease, surgical intervention may be a necessity [4]. 

Initial empiric antibiotic therapy is generally directed against staphylococci and streptococci and typically includes MRSA coverage in those who have previous infections, risk factors, or systemic toxicity. If an individual is immunocompromised, antibiotic coverage should be broad, covering gram positive and negative as well as anaerobic organisms [2, 4]. For suspected mycobacterial disease, treatment is the same as pulmonary tuberculosis. Duration of treatment is variable depending on the complications and organisms involved, with mycobacteria requiring longer treatment courses [9]. 

While tuberculosis itself is common around the world, extrapulmonary manifestations tend to be less common. A study by Wang et al. in 2003 demonstrated that 1.8% of culture positive TB cases (21/1153) in Taiwan were TB myositis cases. However, many of these cases had other manifestations of TB, unlike our patient [3]. The difficulty of diagnosis in our patient stems from her immunocompromised state and the lack of other symptoms of tuberculosis. Isolated presentations of TB as myositis are rare and not widely reported in the literature. 

## 4. Conclusion

Tuberculous pyomyositis should be considered in the differential diagnosis of immunosuppressed patients with fevers and myalgias.

## Figures and Tables

**Table 1:** Laboratory values at presentation to the outside hospital and at presentation to our hospital.

Laboratory values	Presentation to OSH	Presentation to our hospital
Sodium (136–146 mMol/L)	122	133
Potassium (3.5–5 mMol/L)	4.1	4.3
Chloride (98–107 mMol/L)	80	93
Bicarbonate (21–31 mMol/L)	33.3	31
BUN (8–26 mg/dL)	16	12
Creatinine (0.5–1.4 mg/dL)	0.79	0.8
Total protein (6.3–7.7 g/dL)	5.6	4.7
Albumin (3.4–5 g/dL)	2.4	2
Calcium (8.4–10.2 mg/dL)	8.2	
Total bilirubin (0.3–1.5 mg/dL)	0.8	0.5
AST (15–41 IU/L)	26	28
ALT (14–52 IU/L)	36	23
Alkaline phosphatase (38–126 IU/L)	120	101
WBC (3.8–10.6 × 10^9^/L)	22,300	12,100
Hemoglobin (11.6–14.6 g/dL)	12.4	10.3
MCV (82.6–97.4 fL)	86.2	85.3
Platelets (156–359 × 10^9^/L)	406	295
ESR (0–40 mm/hr)		39
CRP (<0.748 mg/dL)		15.7
INR (0.8–1.2)		1.1
